# 

**Published:** 1882-02-20

**Authors:** 


Entered at the Post-Office at Atlanta as second-class matter.

VOL. XII. FEBRUARY 20, 1882. NO. 2.

THE

### SOUTHERN MEDICAL RECORD:

A MONTHLY JOURNAL OF PRACTICAL MEDICINE.

Terms: $2 00 per Annum, in Advance; 4 Copies $6.00; Single Copies 20 Cents.

*"QUICQUID PRÆCIPIES ESTO BREVIS."*

### WHERE TO BUY.

William R. Warner & Co.... 1
Celerina.... 2
Petrolina.... 3
Liebig & Co....4-5
Ahl's Splint Mf'g Co.... 6
A. A. Mellier.... 7
Sharp & Dohme.... 8
T. R. Ripley,.... 9
Johnston's Fluid Beef.... 9
Thomas F. Goode.... 10
Nestle's Milk Food,.... 11
Anglo-Swiss Milk Food,.... 11
Dundas, Dick & Co.... 12
Bedford Springs.... 13
Mellin's Food,.... 14
N Y. Pharmacal Association.... 15
Fellows' Hypophosphites,.... 16
McKesson & Robbins,.... (col. leaves)
Kidder & Laird,....(Inside front cover)
J. Bradfield,....(colored leaves)
Allen & Masser,....(colored leaves)
Reed & Carnrick....(inside back cover.)
Parke, Davis & Co....(outside back cover.)
Parke, Davis & Co....(colored leaves.)
Scott's Emulsion....(colored leaves.)
Listerine....(colored leaves.)

Address R. C. WORD, M.D., Managing Editor, Atlanta, Ga.

